# Fast, Quantitative and Variant Enabled Mapping of Peptides to Genomes

**DOI:** 10.1016/j.cels.2017.07.007

**Published:** 2017-08-23

**Authors:** Christoph N. Schlaffner, Georg J. Pirklbauer, Andreas Bender, Jyoti S. Choudhary

**Affiliations:** 1 Proteomic Mass Spectrometry, Wellcome Trust Sanger Institute, Hinxton, Cambridgeshire CB10 1SA, UK; 2 Centre for Molecular Informatics, Department of Chemistry, University of Cambridge, Cambridge, Cambridgeshire CB2 1EW, UK

**Keywords:** proteomics, genomics, open-source software, annotation, proteogenomics, mapping, visualization, genome browser, large-scale, track hubs

## Abstract

Current tools for visualization and integration of proteomics with other omics datasets are inadequate for large-scale studies and capture only basic sequence identity information. Furthermore, the frequent reformatting of annotations for reference genomes required by these tools is known to be highly error prone. We developed PoGo for mapping peptides identified through mass spectrometry to overcome these limitations. PoGo reduced runtime and memory usage by 85% and 20%, respectively, and exhibited overall superior performance over other tools on benchmarking with large-scale human tissue and cancer phosphoproteome datasets comprising ∼3 million peptides. In addition, extended functionality enables representation of single-nucleotide variants, post-translational modifications, and quantitative features. PoGo has been integrated in established frameworks such as the PRIDE tool suite and OpenMS, as well as a standalone tool with user-friendly graphical interface. With the rapid increase of quantitative high-resolution datasets capturing proteomes and global modifications to complement orthogonal genomics platforms, PoGo provides a central utility enabling large-scale visualization and interpretation of transomics datasets.

## Highlights

• A new open-source tool for mapping peptides to reference genomes
• Visualization of variant peptides, post-translational modifications, and quantitation
• Enables proteogenomic mapping of 25 species from Ensembl genomes
• Provides faster, scalable, and accurate mapping of peptides and associated features

## Graphical Abstract

**Figure:**
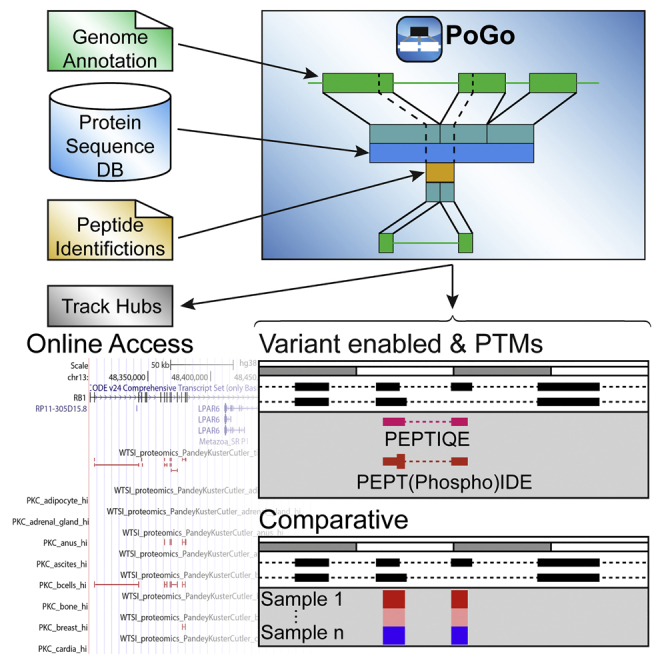

## Teaser

We have developed a fast, scalable, and free open-source tool to map peptides identified through mass spectrometry onto reference genomes. It provides extended functionality, supporting representation of single-nucleotide variants, post-translational modifications, and quantitative features. The tool has been integrated in established frameworks and is available as a standalone version with user-friendly graphical interface.

## Introduction

Mass spectrometry (MS) and next-generation sequencing technologies have vastly improved our understanding of the cross-talk between genome, transcriptome, and proteome and contribute to a better understanding of the variations between healthy and disease states. Examples are the identification of new therapeutic target kinases in breast cancer (Mertins et al., 2016) and detection of differentially regulated pathways and functional modules potentially enabling patient stratification in ovarian cancer to inform therapeutic management (Zhang et al., 2016).

**Figure 1:**
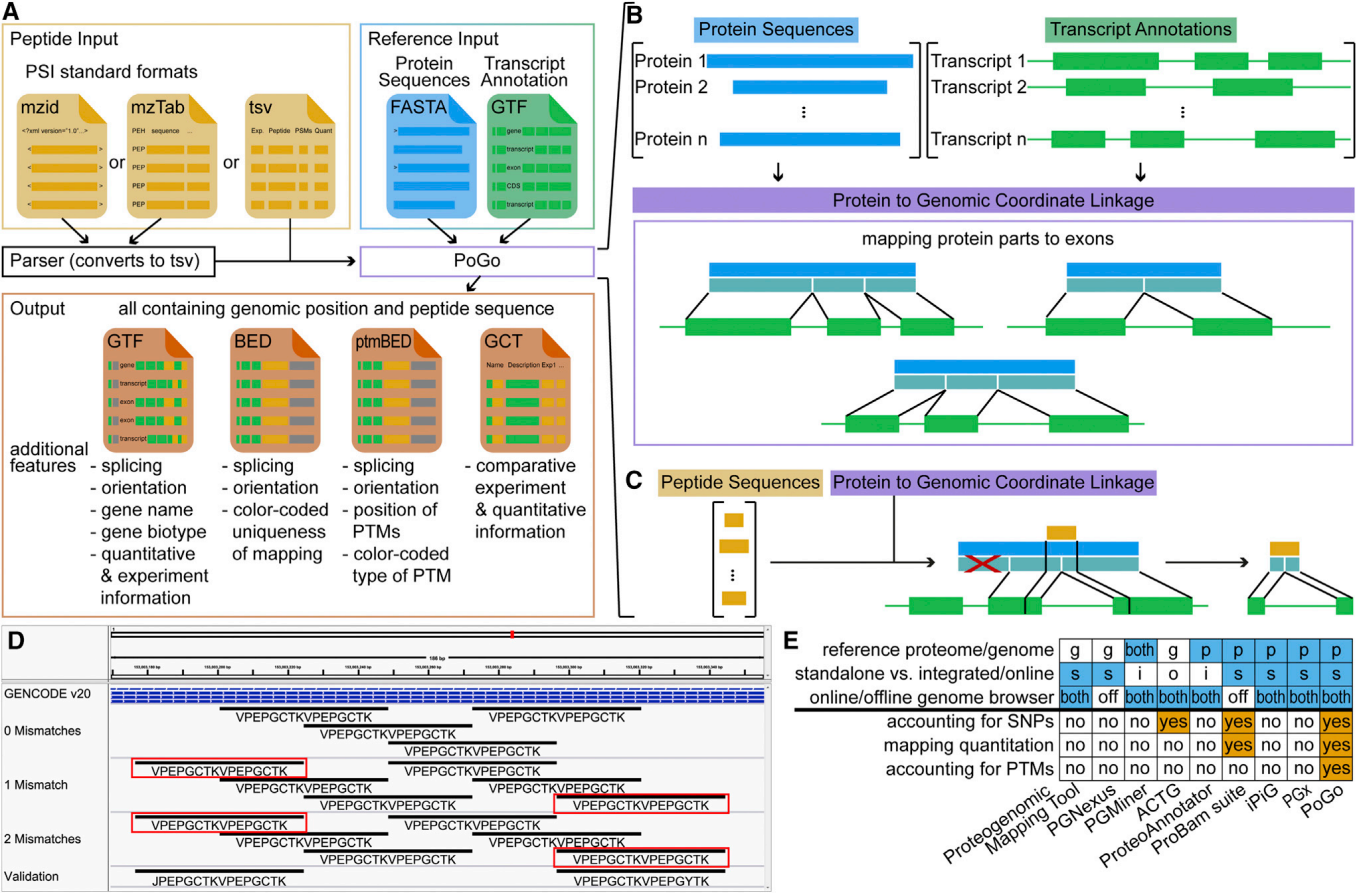
Schema of the PoGo Algorithm for Mapping Peptides through Proteins to Genomic Loci (A) Transcript annotation (GTF) and translated sequences (FASTA) form the reference input for PoGo. Standardized proteomics formats are converted into proprietary tab-separated format with minimal peptide information. All four output formats of PoGo contain genomic alignment supplemented with specifications for uniqueness of mappings, quantitative information, and post-translational modifications. (B) Annotated protein coding transcripts in GTF format and respective translated protein sequences in FASTA format are integrated by PoGo through intermediate coordinates (turquoise), representing the exonic structure of the transcript within the protein. (C) Peptides, identified through searching mass spectrometry data against the protein sequence database, are mapped against the proteins (see also Figure S4). The position within the proteins then allows retrieval of overlapping coding exons and enables the calculation of the exact genomic coordinates. (D) Example mappings of PoGo for the overlapping repeat peptide VPEPGCTKVPEPGCTK in a genome browser (0 mismatches). Application of PoGo allowing for up to two mismatches results in identification of two additional repeats (1 and 2 mismatches, red boxes; see also Figures S1, S5, and S6). The additional mappings of the initial peptide sequence were validated through peptides of the exact sequence identified in the same mass spectrometry experiment (validation). Leucine (L) and isoleucine (I) are substituted through their common single-letter code “J.” (E) Comparison of different peptide-to-genome mapping tools with regard to reference sequence type, integration into frameworks, support of online and offline genome browsers (blue). Additional features (orange) indicate the superior performance of PoGo over other tools.

Substantial advances in MS technologies enable more complete identification and quantification of proteomes, making these data more comparable with transcriptomics (Aebersold and Mann, 2016). Tools to readily visualize proteomics with corresponding RNA-sequencing data on a reference genome are now increasingly indispensable. Numerous approaches have been implemented such as Proteogenomic Mapping Tool (Sanders et al., 2011), PGNexus (Pang et al., 2014), PGMiner (Has et al., 2016), ACTG (Choi et al., 2016), ProteoAnnotator (Ghali et al., 2014), ProBamSuite (Wang et al., 2016), iPiG (Kuhring and Renard, 2012), and PGx (Askenazi et al., 2016). Key attributes such as mapping reference (proteome or genome), grade of integration with other proteomics tools, and support of online and offline browsers through output formats distinguish the approaches (Figure 1E). While iPiG, for example, heavily relies on the annotation format used for UCSC genes, PGx uses sample-specific protein sequence databases derived from RNA-sequencing experiments and corresponding genomic coordinates. Both tools, however, require reformatting of a reference genome annotation in order to enable their mapping. Reference genome annotation is frequently updated and reformatting new versions by users is a recurrent source of errors that propagate to the proteogenomic mapping. Consequentially, reformatting reference genome annotation prevents efficient and accurate use of these tools.

## Results

**Figure 2:**
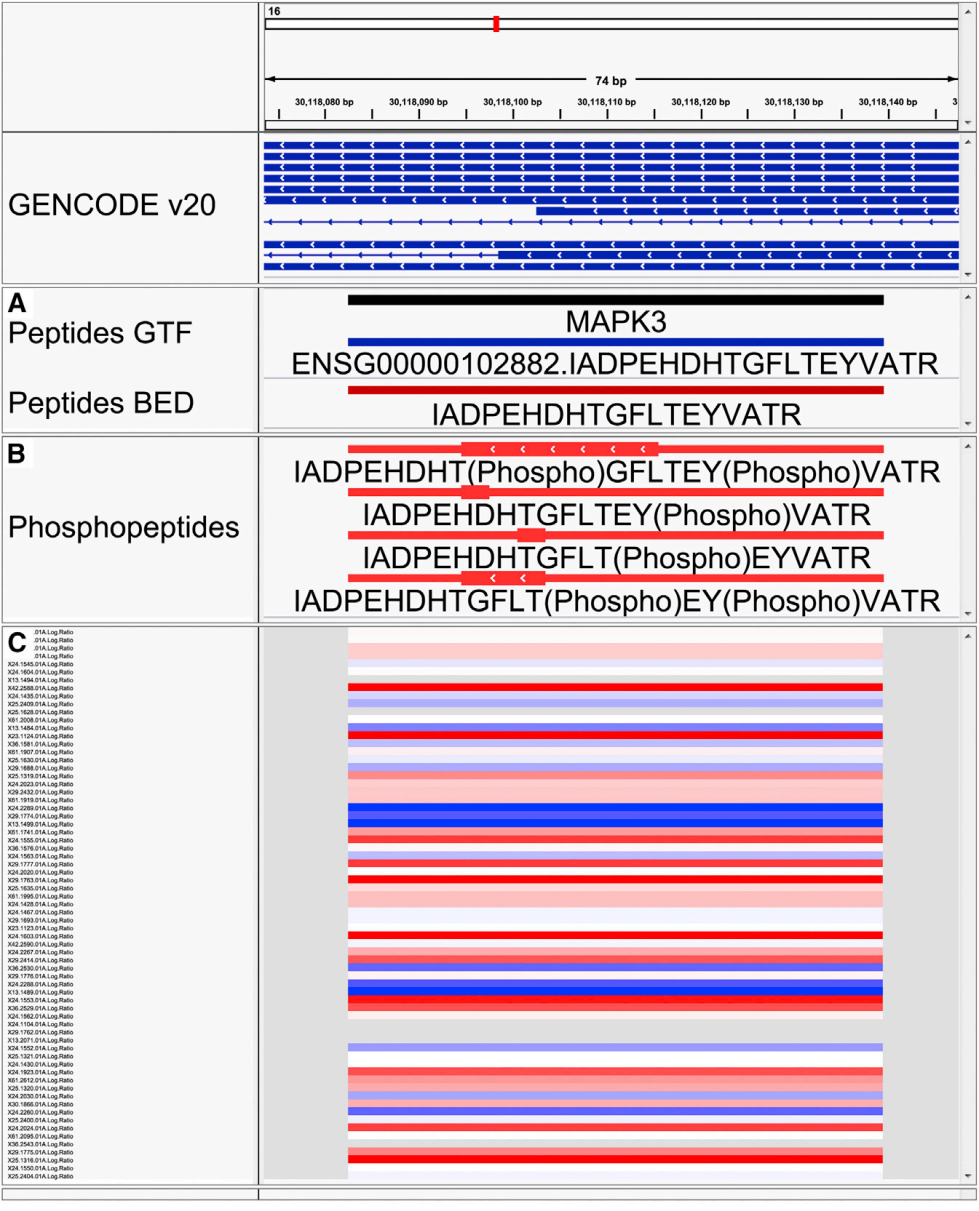
Visualization in the Integrative Genomics Viewer of Different PoGo Output Formats for the Peptide IADPEHDHTGFLTEYVATR within the *MAPK3* Gene Genomic coordinates are shown at the top as the x axis. GENCODE (v20) annotations of transcripts are indicated in blue. (A) In addition to the genomic location of the peptide, the GTF format also holds other information, such as the gene name and gene identifier, while the BED output visualizes uniqueness of the mapping across the genome. Here, the red color indicates unique mapping to a single transcript of *MAPK3*. (B) Genomic loci of post-translational modifications within a peptide; here, phosphorylation identified by brackets in the sequence, are depicted by thick blocks spanning from the first and last modification site. The red color in this output format indicates the presence of phosphorylation (see also Table S1). (C) View of log2-fold changes mapped for the example peptide to the genomic location across 69 ovarian cancer samples (y axis). High values are shown in red while blue indicates low log2 ratios (see also Figure S7).

We developed PoGo to allow direct mapping to reference annotations and improve the speed and quality of mapping. PoGo leverages the annotated protein coding sequences (CDS) together with a reference protein sequence database (protein-DB) to map peptides to their genomic loci. Firstly, PoGo maps the genomic coordinates of CDSs onto the protein (Figure 1B), thereby connecting the protein sequences to the genomic coordinate space. Database search tools enable peptides to be identified from MS using a protein-DB (Perez-Riverol et al., 2014). By using the PoGo-indexed database, genomic coordinates of a peptide are retrieved based on the peptide's position within the protein (Figure 1A and STAR Methods). PoGo further takes advantage of distinct attribute columns of the output file formats, such as color, to indicate the uniqueness of a peptide across the genome, to show positions of post-translational modifications, to allow quantitative comparison between multiple samples and conditions linking this information to transcripts and genetic loci (Figure 2 and STAR Methods). The main genome browsers, Ensembl (Kent et al., 2002), UCSC (Yates et al., 2016), and BioDalliance (Down et al., 2011), however, have file size limits for direct upload insufficient for large-scale proteogenomics. Our track-hub generator application, therefore, enables seamless online visualization directly from PoGo output and is crucial for open-access proteomics of large datasets.

We first evaluated PoGo's performance on large-scale datasets using the proteogenomic reanalysis of the draft human proteome maps (Wright et al., 2016). We used the filtered high stringency level set comprising ∼3 million peptides across 59 adult and fetal tissues (233,055 unique sequences). The mappings were derived from the gene annotation set and protein coding translation sequences for GENCODE (release 20) (Wright et al., 2016) as GTF and FASTA files. All tools were run with standard parameter settings and evaluated based on speed, memory usage, and number of unique and correct mappings. PoGo (94 s) was 6.9 and 96.4 times faster than PGx (651 s) and iPiG (memory error after 9,064 s), respectively, and required 20% less memory compared with PGx (9.7 GB and 11.9 GB, respectively). These data show a major improvement in speed and memory usage in addition to application with a readily available reference annotation.

In total, 89% of mappings are common between PoGo and PGx. The 10.5% uniquely reported by PGx could be attributed to false assignments that were resolved after shifting their frame to insure correct alignment with the genome. PoGo resulted in 89 completely unique mappings, 72 of these were correct but incomplete mappings to the start and end of protein sequences that can be attributed to incompletely annotated transcripts (CDS start/end not found). In addition, 17 unique mappings correspond to alternative splicing, immunoglobulin genes, and multiple overlapping mappings in a repeat region. For example, the peptide VPEPGCTKVPEPGCTK (missed cleavage between repeats of eight amino acids) was mapped by PGx as two consecutive loci in the *SPRR3* gene (Figure S1). PoGo, on the other hand, mapped the sequence four times with the repeats overlapping each other (Figure 1C). These repeat mappings demonstrate the enhanced quality of mappings through our tool; all occurrences of peptides within the translated protein coding sequences are correctly identified.

The fast and diverse mapping capabilities of PoGo, as shown above, prompted the current integration of the algorithm into the PRIDE (Vizcaino et al., 2013) tool suite and soon into the OpenMS framework (Wright et al., 2016). This dataset also exemplifies the growing need to handle large numbers of peptides. Therefore, we have generated tissue track hubs, which are web-accessible directories of genomic data for visualization of a large number of genome-wide datasets at two different significance thresholds from the draft human proteome maps, allowing identification of genes and transcripts unique to single tissues. The scaffolding protein CASS4, for example, was found only in platelets tissue track represented in the high-significance filtered hub (Figure S2). The peptide support for all splice junctions of *RBP3* was only identified with high confidence in the retina dataset. The corresponding genomic region is depicted in Figure S3.

The large number of single-nucleotide variants in individuals can affect the protein sequences and hinder identification of peptides through database searching against a reference genome (Vizcaino et al., 2013). Uniquely compared with other tools, PoGo is able to account for up to two non-synonymous variants, resulting in single amino acid substitutions, in its mapping (Figure S4). Application with the draft human proteome maps allowing one and two variants resulted in a 1.5- and 60.8-fold increase in runtime (Figure S5). Unique mappings to single transcripts were reduced by 5.1% and 15.9% while the number of peptides belonging to multiple genes increased exponentially by 220.9% and 3,175.2% (Figures S5 and S6). The mapping of additional repeats of the sequence VPEPGCTK following application with mismatches were validated through identified peptides in the sample (Figure 1C). This highlights the added value to PoGo for mapping peptides to genomic loci with potential single-nucleotide variants.

To demonstrate additional PoGo functionalities, we chose the phosphoproteome of high-grade serous ovarian cancer with isobaric labeling of 96 tumor samples, identifying 13,646 unique peptides with annotated phosphorylation sites (19,156 phosphopeptides) (Zhang et al., 2016). PoGo mapped 13,617 peptides to 15,944 genomic loci in 66.9 s; these could not be mapped by PGx and iPiG due to phosphorylation annotation in the peptide sequences. Only a small fraction, 0.2%, of the peptides could not be mapped due to sequence differences of the originating proteins between RefSeq and GENCODE databases. Compared with the other tools, PoGo was able to use the annotated post-translational modifications and color code them (Table S1) resulting in mappings for 99.8% of the phosphopeptides with their respective localized phosphorylation sites on the reference genome (Figure S7).

PoGo also integrates peptide quantitation with genomic loci through the GCT file format. This allows comparative visualization of multiple samples in the Integrative Genomics Viewer (Thorvaldsdottir et al., 2013) and enables downstream quantitative analysis. The log2-fold changes of phosphopeptides between all 69 ovarian cancer samples and the pooled reference were mapped with PoGo (Figure S7). As an example, *MAPK3* identified with multiple phosphorylated sites in a single peptide and the associated fold changes across samples are shown in Figure 2. To our knowledge, PoGo is the only tool directly integrating quantitative information for peptides with genomic coordinates.

## Discussion

Our data show that PoGo represents a major advance for peptide-to-genome mapping, making it a cornerstone component of proteogenomics workflows. Although the examples used here focus on human tissue and cancer cell lines, PoGo can be applied to any proteomic study for which annotation of coding sequences in GTF format and translated sequences in FASTA format are available. The additional functionalities, such as allowing up to two non-synonymous single-nucleotide variants, mapping of post-translational modifications, and integration of quantitation, distinguish it from other tools. Semi-standardized file formats commonly used in genomics for input and output, as well as the scalability for large datasets, make PoGo an indispensable component of small- and large-scale multi-omics studies. The current integration into the PRIDE tool suite and our track-hub generator application promote open-access proteogenomics, supporting studies focusing on integration of gene, protein, and post-translational modification expression (Alvarez et al., 2016) in the future. PoGo has been developed to cope with the rapid increase of quantitative high-resolution datasets capturing proteomes and global modifications. Integration of orthogonal genomics platforms with these datasets through PoGo will be valuable for large-scale analysis such as personal variation and precision medicine studies.

## STAR★Methods

### Key Resources Table

**Table:** 

REAGENT or RESOURCES	SOURCE	IDENTIFIER
**Deposited Data**		

Ensembl Human Genome Primary Assembly, release 76	Yates et al. (2016)	ftp://ftp.ensembl.org/pub/release-76/fasta/homo_sapiens/dna; RRID: SCR_002344
GENCODE, release 20	Wright et al. (2016)	ftp://ftp:sanger.ac.uk/pub/gencode/Gencode_human/release_20; RRID: SCR_014966
Reanalysis of draft human proteome maps	Wright et al. (2016)	PRIDE: PXD002967
Phosphopeptide summary, Ovarian Cancer, CPTAC, Phase 2	Zhang et al. (2016)	https://cptc-xfer.uis.georgetown.edu/publicData
Draft human proteome maps track hubs	this paper	ftp://ngs.sanger.ac.uk/production/proteogenomics

**Software and Algorithms**		

PoGo website	This paper	http://www.sanger.ac.uk/science/tools/PoGo
PoGo	This paper	https://github.com/cschlaffner/PoGo
PoGo GUI	This paper	https://github.com/cschlaffner/PoGoGUI
FileConverter	This paper	https://github.com/cschlaffner/FileConverter
Track-Hub Generator	This paper	https://github.com/cschlaffner/TrackHubGenerator
Perl 5.16.2	The Perl Programming Language	https://www.perl.org
R 3.3.1	The R project	https://www.r-project.org
GNU C++ compiler (gcc) 6.2.0	GNU Compiler Collection	https://gcc.gnu.org
Microsoft C/C++ Optimizing Compiler 18.00.31101	Visual Studio Express 2013	https://www.microsoft.com/en-gb/download
PGx	Askenazi et al. (2016)	https://github.com/FenyoLab/PGx
iPiG	Kuhring and Renard (2012)	https://sourceforge.net/projects/ipig
fetchChromSizes.sh	UCSC Genome Bioinformatics	http://hgdownload.soe.ucsc.edu/admin/exe/linux.x86_64
bedToBigBed	UCSC Genome Bioinformatics	http://hgdownload.soe.ucsc.edu/admin/exe/linux.x86_64
Integrative Genomics Viewer (IGV) v2.3.68	Thorvaldsdottir et al. (2013)	http://software.broadinstitute.org/software/igv/download
UCSC Genome Browser	UCSC Genome Bioinformatics	http://genome.ucsc.edu
Ensembl Genome Browser	Ensembl Archives	http://aug2014.archive.ensembl.org
BioDalliance Genome Browser	GENCODE	http://www.gencodegenes.org

### Contact for Reagent and Resource Sharing

Please contact the Lead Author, Christoph Schlaffner (christoph.schlaffner@sanger.ac.uk), further information and requests for resources and reagents.

### Method Details

#### Implementation of PoGo

PoGo is a multi-sample peptide-to-genome mapping tool taking as input tab delimited lists of peptides identified through mass spectrometry (MS) with associated number of peptide-to-spectrum matches (PSMs), quantitative value and sample identifier. PoGo also requires a reference genome annotation in the General Transfer Format (GTF) and translated protein coding sequences in FASTA format as input. The genomic coordinates of annotated coding sequences are mapped onto their respective protein sequences. Peptides identified through MS are then mapped against protein sequences accounting for up to two mismatches. The genomic coordinates for each peptide are calculated based on their position within the proteins. Each mapped peptide is additionally assigned the associated sample identifier as well as the number of PSMs and the quantitative value. Furthermore, post-translational modifications annotated in the peptide sequence are mapped to their respective genomic coordinates and color coded for the type of modification.

A separate FileConverter implemented in java supports standardized PSI file formats such as mzIdentML and mzTab through use of ms-data-core-api. (Perez-Riverol et al., 2015) Furthermore, the file converter is integrated into a java implemented graphical user interface (GUI) for PoGo enabling non-bioinformaticians to map peptides to reference genomes.

##### Connecting Proteins with Genomic Coordinates

PoGo requires protein sequences and gene annotations in FASTA and GTF format, respectively. Protein sequences have to be connected to genes and transcripts through type specific identifiers (IDs). For each protein sequence lines from the GTF file containing the transcript ID and feature-type CDS (coding sequence) are extracted. The order of exons per transcript starts with the first exon in the sequence reflecting the reading direction during translation, regardless of the strand, resulting in a reverse order of genomic coordinates for transcripts on the reverse strand. This way protein sequences and the exons match directionality. The exonic structure is mapped onto the protein sequence through construction of protein exons. Let a transcript T be a set of exons t_1_, t_2_, … t_n_ where n is the number of exons and each exon t contains the chromosome identifier, the start and end positions within the chromosome, S_t_ and E_t_ respectively, the strand on which the transcript is annotated. The corresponding protein P is defined as a set of protein exons p_1_, p_2_, … p_n_, where each protein exon p contains the start and end positions, s_p_ and e_p_ respectively, within the protein sequence so that the protein is mapped onto the transcript as $f:P\to T,\;p_{i}\to t_{i}$. For each protein in the FASTA file a map of protein exons to genomic exons is generated in PoGo.

To account for frame shifts between genomic exons t_i_ and t_i+1_ each protein exon p also holds information about the number of base pairs (bp) contributing to the codon of the first (N-term) and last (C-term) amino acid as offsets O={1,2,3}. In general, the N-term offset at the beginning of a protein defined as $O\left(p_{1}\left(N\_term\right)\right)=3$ resulting in $O\left(p_{n}\left(C\_term\right)\right)=3$ for complete annotations of coding transcripts. In instances where the annotation is missing a start or end codon the offsets may vary and is identified through the annotated frame. C-term offsets $O\left(p_{i}\left(C\_term\right)\right)$ for each protein exon p are calculated based on the length of the genomic exon $L\left(t_{i}\right)$ and the offset of the N-term $O\left(p_{i}\left(N\_term\right)\right)$ so that $O\left(p_{i}\left(C\_term\right)\right)=X=L\left(t_{i}\right)\;mod\;3-O\left(p_{i}\left(N\_term\right)\right)+3$ with the exception $O\left(p_{i}\left(C\_term\right)\right)=X\;mod\;3$ for X>3. N-term offsets of following protein exons $O\left(p_{i+1}\left(N\_term\right)\right)$ are calculated so that $O\left(p_{i}\left(C\_term\right)\right)+O\left(p_{i+1}\left(N\_term\right)\right)\;mod\;3=0$.

##### Identifying Proteins of Origin for Peptides

To allow fast lookup of proteins containing any given peptide PoGo creates a dictionary of words with length k (k-mer) overlapping by k-1 amino acids from the protein sequences in the FASTA input. Associated with each k-mer is a list of protein entries containing the associated protein with identifiers and the start position of the k-mer in the sequence. The dictionary is designed to consider leucine (L) and isoleucine (I) as equal through substitution with the shared one letter code ‘J’ as they are not distinguishable in MS. Peptides identified through MS are retrieved from the input file and searched against the dictionary. Thereby PoGo allows imperfect matching with up to 2 amino acid substitutions (mismatches m) to also identify proteins with potentially underlying non-synonymous single nucleotide variants. For peptides shorter than (m+1)×k residues only the first word of length k is used and all combinations with m amino acid substitutions are generated. Each new word is looked up in the dictionary. Peptides longer than (m+1)×k are split into consecutive k-mers and searched in the dictionary. At most m consecutive k-mers can contain amino acid substitutions leaving one word without any substitutions allowing for perfect matching in the look-up table. The presence of the peptide in each found protein then is validated taking into account the number of mismatches. The gene and transcript identifiers and the respective start position within each protein are retrieved.

##### Retrieving Genomic Coordinates for Peptides

Peptides with associated gene and transcript identifiers and the start positions within each protein are used to calculate the genomic coordinates. The length of the peptide sequence A with start position s_A_ in protein P is used to calculate the end position e_A_. To calculate the genomic coordinates for the peptide first the overlapping protein exons p are obtained so that $P\left(A\right)=\left\{xɛP|s_{x}\leq s_{A}\leq e_{x}\;v\;s_{x}\leq e_{A}\leq e_{x}\right\}$. Through the mapping of protein exons to genomic exons PoGo can now retrieve the genomic exons for the peptide sequence A through P(A)→T(A). The genomic coordinates then are calculated as start $S_{A}=S_{E}+dS_{A}$ and end $E_{A}=S_{E}+dE_{A}$ if the gene is on the forward strand or start $S_{A}=S_{E}-dS_{A}$ and end $E_{A}=S_{E}-dE_{A}$ if on the reverse strand with $dS_{A}=\left(s_{A}-s_{P}-1\right)\times 3+O\left(P\left(N\_term\right)\right)$ and $dE_{A}=\left(e_{A}-s_{P}\right)\times 3+O\left(P\left(N\_term\right)\right)-1$ denoting the distance of the genomic start and end of the peptide, respectively, from the genomic start position S_E_ of the genomic exon E.

##### Mapping Post-translational Modifications

Besides mapping peptides, PoGo is also capable of mapping post-translational modifications (PTMs) onto the genome. Post-translational modifications are commonly annotated in the peptide sequence through round brackets containing the PSI (Proteomics Standards Initiative) name of the modification following the modified amino acid. With the position of post-translational modifications in the peptide sequence, start s_PTM_ and end e_PTM_, the mapping of the underlying peptide to the genome the above equations to calculate the genomic positions are adjusted: $dS_{PTM}=\left(s_{A}+s_{PTM}-s_{P}-1\right)\times 3+O\left(P\left(N\_term\right)\right)$ and $dE_{PTM}=\left(s_{A}+e_{PTM}\right)\times 3+O\left(P\left(N\_term\right)\right)-1$. Different types of PTMs are mapped separately and color coded in the output while multiple occurrences of the same PTM type, e.g. phosphorylation, within a single peptide are combined into a single mapping using the first and last PTM sites.

##### Adding Quantitative Information

To allow visualization of quantitative information for peptides on a genome, PoGo records this type of information. Peptide and sample pairings may only occur once in the input file uniquely identifying a quantitation value. PoGo stores the tuples of sample identifier, quantitative value and the number of peptide to spectrum matches (PSMs) for each peptide. This information is used in the different output formats to allow comparative analysis.

##### Generating Different Output Formats

PoGo generates output in three formats commonly used in genomics. The first and central output format of PoGo is BED. This format stores each mapped peptide as a single line of twelve tab delimited columns. Besides chromosome coordinates, the peptide sequence, strand as well as start and end coordinates of a thick block the start positions and lengths of peptide blocks mapping to genomic exons are included. Additionally, BED files support individual coloring of each feature. PoGo utilizes this in two different forms. Firstly, in the general peptide centric output of PoGo peptides are colored based on their uniqueness within the genome. Peptides unique to a single transcript are colored in red while peptides shared between multiple transcripts of a single gene are shown in black. Peptides mapping to multiple genes are indicated by their grey color. Secondly, PoGo also generates a separate BED file for peptide forms with post-translational modifications. In this instance the thick block element is used to indicate the position of the post-translational modification. Two or more modifications of the same type within a single peptide sequence are collapsed to indicate the range between the first and last modification site. The coloring of the uniqueness per peptide in the genome is substituted to accommodate color coding of post-translational modifications.

The second file format supported by PoGo for mapped peptides is the general transfer format (GTF). PoGo redefines some of the feature types to accommodate mapping of peptides. The feature type ‘transcript’ is used to indicate a mapped peptide while the feature type ‘exon’ indicates the concrete mapping of the peptide to underlying genomic exons. PoGo additionally stores information such as the gene identifier, name and biotype for the gene as well as the number of peptide-to-spectrum matches (PSMs) and quantitative values for each sample in which the peptide was identified.

For comparative or quantitative analysis PoGo generates the output format GCT which can be visualized in the Integrative Genomics Viewer (IGV). (Thorvaldsdottir et al., 2013) This third format is similar to a matrix with rows identifying a peptide with genomic mapping and columns identifying a sample. Each cell holds the quantitative values associated with the peptide and the sample given in the input file.

#### Testing

##### Human Tissue Data

High-resolution MS data from 59 fetal and adult human tissues were used for the validation of PoGo. The raw data of these draft human proteome maps were generated by the Pandey lab (Kim et al., 2014), the Kuster lab (Wilhelm et al., 2014), and Cutler lab. (Desiere et al., 2006) All three datasets were combined and reprocessed by Wright et al. (2016) The data were retrieved in a tab delimited format combining all results from mzid files available from PRIDE Archive. (Vizcaino et al., 2013) Identifications were filtered to the highest stringency level described in Wright et al. (2016) for identification of novel coding regions (q-value ≤ 0.01 (1% FDR), a PEP of ≤ 0.01, peptide length between 7 and 29 residues, full tryptic peptides, a maximum of two missed cleavages).

##### Phosphoproteomic Ovarian Cancer Data

We applied PoGo to isobaric labelled phosphoproteome data from an ovarian tumor study comprising 69 samples. (Zhang et al., 2016) Phosphopeptides with associated iTRAQ quantitation were downloaded as tab separated file from https://cptac-data-portal.gorgetown.edu. Lower case characters (s, t and y) in the peptide sequence showing phosphorylation were substituted by upper case characters followed by the PSI name of phosphorylation in brackets.

##### Selected Peptides for Feature Testing

For testing features of PoGo a total of 14 peptides were selected from the above datasets. These peptides include single exon peptides, peptides spanning up to 2 splice junctions, mapping to multiple genes and repeats. Additionally, multiply phosphorylated peptides are included for PTM mapping. Compiled versions for Windows, Linux/Unix and Mac as well as the graphical user interface are available alongside this test dataset and detailed step by step instructions in Data S1.

##### Reference Data and PoGo Settings

The annotation of human genes in GTF format and the corresponding protein coding sequence translation as FASTA files were downloaded for GENCODE v20 (Wright et al., 2016) from http://www.gencodegenes.org. Gene and transcript identifiers were set as “ENSG” and “ENST” for genes and transcripts, respectively, followed by 11 digits and the word length for k-mers was set to 5 amino acids. For post-translational modifications 10 biologically relevant types were chosen for easy discriminability of the color code (Table S1).

##### Application of Tools for Comparison

For the human tissue and the ovarian cancer phosphoproteome data PoGo's performance was compared against PGx (Askenazi et al., 2016) (downloaded from https://github.com/FenyoLab/PGx) and iPiG (Kuhring and Renard, 2012) (downloaded from https://sourceforge.net/projects/ipig/), two standalone tools available to map peptides to their corresponding genomic coordinates. Each dataset was formatted using in-house scripts in R and perl to fit the required input format for each tool. Each program was run using default parameters and the minimum number of required input files. Time and memory usage for tool comparisons were measured on Ubuntu 12.04 using an Intel Xeon CPU E5-2680 v2 with 2.80 GHz and 100 GB random access memory. Comparison of the effect of allowing mismatches in PoGo mapping were run on a computer cluster running Linux 64bit with CPU type 2x 2.1 GHz 16 core AMD 6378 and 256 GB memory.

#### Generating of Track Hubs from PoGo Output

Track hubs were generated to visualize different aspects of the human proteome maps. The data was filtered to two stringency levels resulting in two sets. The first result set was filtered to a standard significance (q-value of ≤ 0.01 (1% FDR), a PEP of ≤0.05 and a minimum peptide length of 7 residues) while the highest stringency level mentioned in Wright et al. (2016) (q-value ≤ 0.01 (1% FDR), a PEP of ≤ 0.01, peptide length between 7 and 29 residues, full tryptic peptides, a maximum of two missed cleavages) was applied to the second set. Additionally, each set was split into subsets for individual tissues, resulting in 60 files per set. PoGo was run with default parameters using the property of passing a comma separated list of input files to be mapped separately. The Track-Hub Generator application then was run using the 60 output files in BED format to generate two track hubs; one for each significance level filter. Folders and files required for track hubs are generated automatically. The script ‘fetchChromSizes.sh’ and tool ‘bedToBigBed’ from UCSC (both downloaded from http://hgdownload.cse.ucsc.edu) (Kent et al., 2010) are used in the Track-Hub Generator to create binary files from the original BED files used for track hubs. The generated track hubs are accessible through ftp and http via http://www.sanger.ac.uk/science/projects/proteogenomichubs (see Figures S2 and S3).

### Quantification and Statistical Analysis

#### Comparison between Tool Outputs

To compare the mappings between the tools, instances were marked as equal when chromosome name, start and end positions, the exon starts and lengths as well as the peptide sequence were the same using the merge function in R (https://www.r-project.org). Frameshifts then were identified amongst unique mappings per tool through shifting start and end positions by up to two base pairs and comparing those to the consensus mappings. Remaining unique mappings of the tools then were examined manually by comparing the peptide sequence to the translated sequence of the respective genomic coordinates in the IGV browser. (Thorvaldsdottir et al., 2013).

### Data and Software Availability

PoGo executables for Windows, Mac and Linux as well as PoGo GUI and FileConverter for PoGo are available from here: http://www.sanger.ac.uk/science/tools/pogo.

PoGo source code in C++ is available via github: https://github.com/cschlaffner/PoGo.

PoGo GUI source code in java is available via github: https://github.com/cschlaffner/PoGoGUI.

FileConverter for PoGo source code in java is available via github: https://github.com/cschlaffner/FileConverter.

The Track-Hub Generator application is available here: http://www.sanger.ac.uk/science/tools/trackhub-generator.

Track-Hub Generator is also available on github: https://github.com/cschlaffner/TrackHubGenerator.

The generated track hubs for high and standard significance are accessible in the Sanger Institute's website: http://www.sanger.ac.uk/science/data/proteomics-trackhubs.

## Author Contributions

C.N.S. conceived and designed the algorithms, implemented the genomic mapping algorithm, performed comparisons with other algorithms, and wrote the manuscript; G.J.P. implemented the protein identification algorithm; A.B. and J.S.C. supervised the work and wrote the manuscript.

## Supplemental Information

Document S1. Figures S1–S7 and Table S1

Data S1. PoGo Test Procedures and Files, Related to STAR Methods

Document S2. Article plus Supplemental Information
