# Supplementary material for: Fast, Quantitative and Variant Enabled Mapping of Peptides to Genomes
Source: Cell Syst. 2017 Aug 23;5(2):152–156.e4. doi: 10.1016/j.cels.2017.07.007 (PMC5571441; doi:10.1016/j.cels.2017.07.007)
Supplement: Document S1. Figures S1–S7 and Table S1 [file mmc1.pdf]

Cell Systems, Volume *5*

Supplemental Information

Fast, Quantitative and Variant Enabled Mapping of Peptides to Genomes

Christoph N. Schlaffner, Georg J. Pirklbauer, Andreas Bender, and Jyoti S. Choudhary

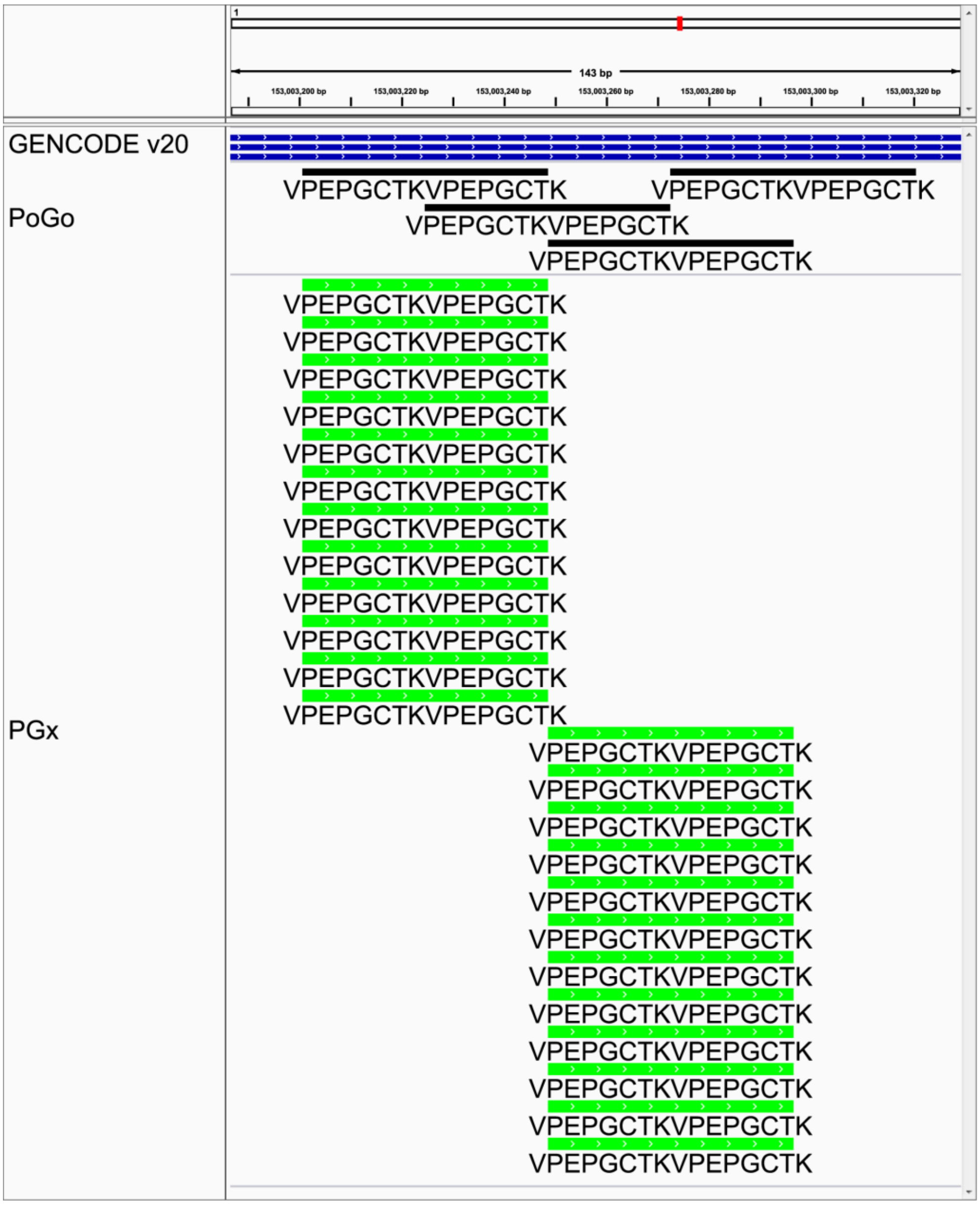

Figure S1. Mapping to repeat region, Related to Figure 1. Visualization in IGV of peptide mappings in a genome browser with genomic coordinates shown at the top as x-axis. The peptide 'VPEPGCTKVPEPGCTK' with missed cleavage between two repeats of 8 amino acids within the gene SPRR3 (GENCODE (v20) annotation shown in blue) is mapped to four overlapping loci (black) while PGx only maps it to two consecutive loci (green). Furthermore, PoGo only maps each peptide once to the same locus. PGx, however, maps all occurrences within the input set to each genomic position.

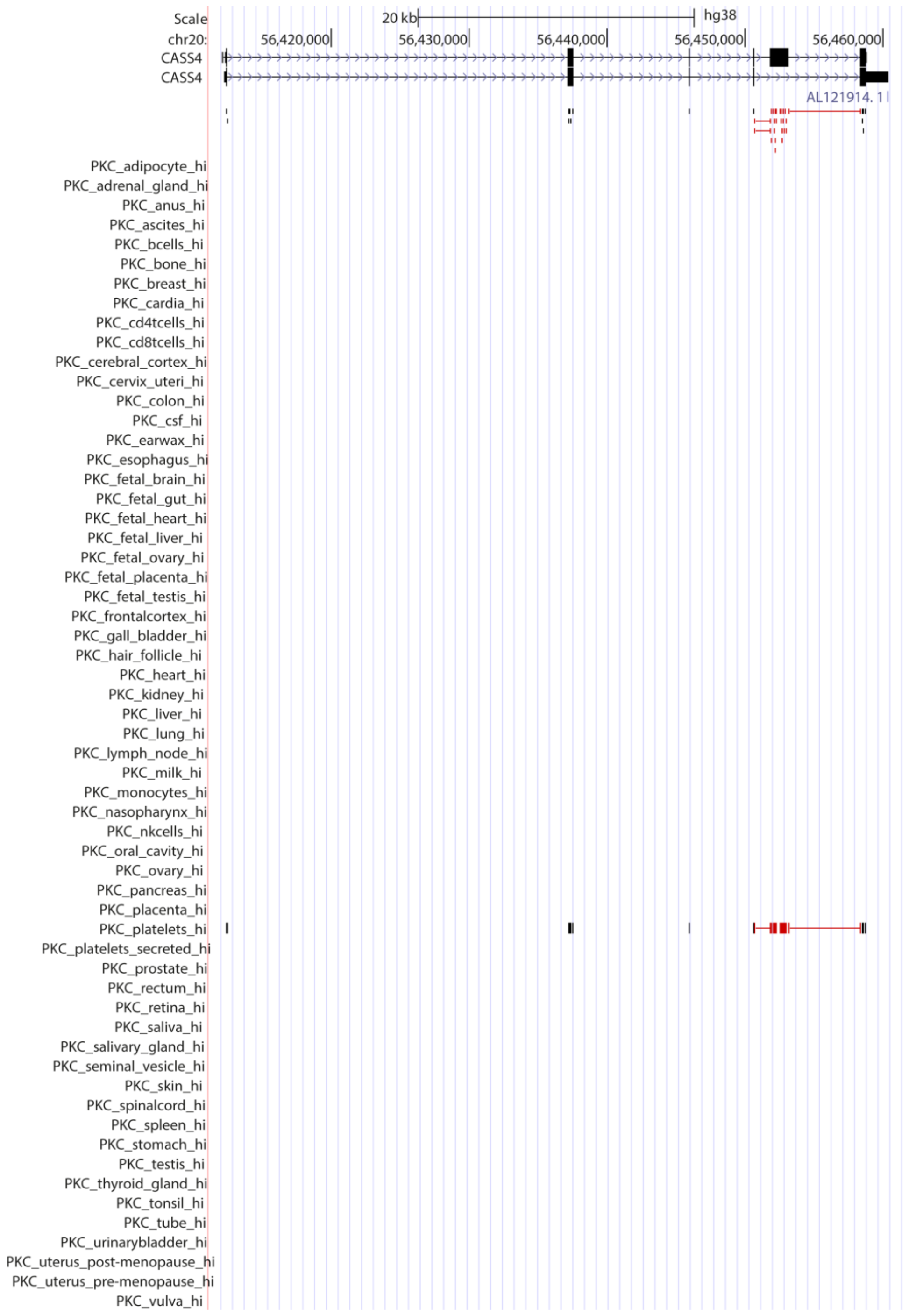

Figure S2. Track-hub visualization for peptides of CASS4, Related to STAR Methods. Visualization of track-hub generator output for the reanalyzed draft human proteome maps in the UCSC genome browser for the genomic region of *CASS4*. Genomic coordinates are shown as x-axis while tissues within the dataset represent the y-axis. GENCODE (v20) annotation of two transcripts is shown in black at the top. Peptides identified in the whole dataset are shown underneath in red, representing unique mapping to a single transcript, and black, indicating unique mapping to the gene. Peptides identified within single tissues are shown below. All peptides identified in the region were only found in platelets (red and black bars in the lower third of screenshot). The protein is involved in tyrosine kinase-based signaling related to cell adhesion and spreading.

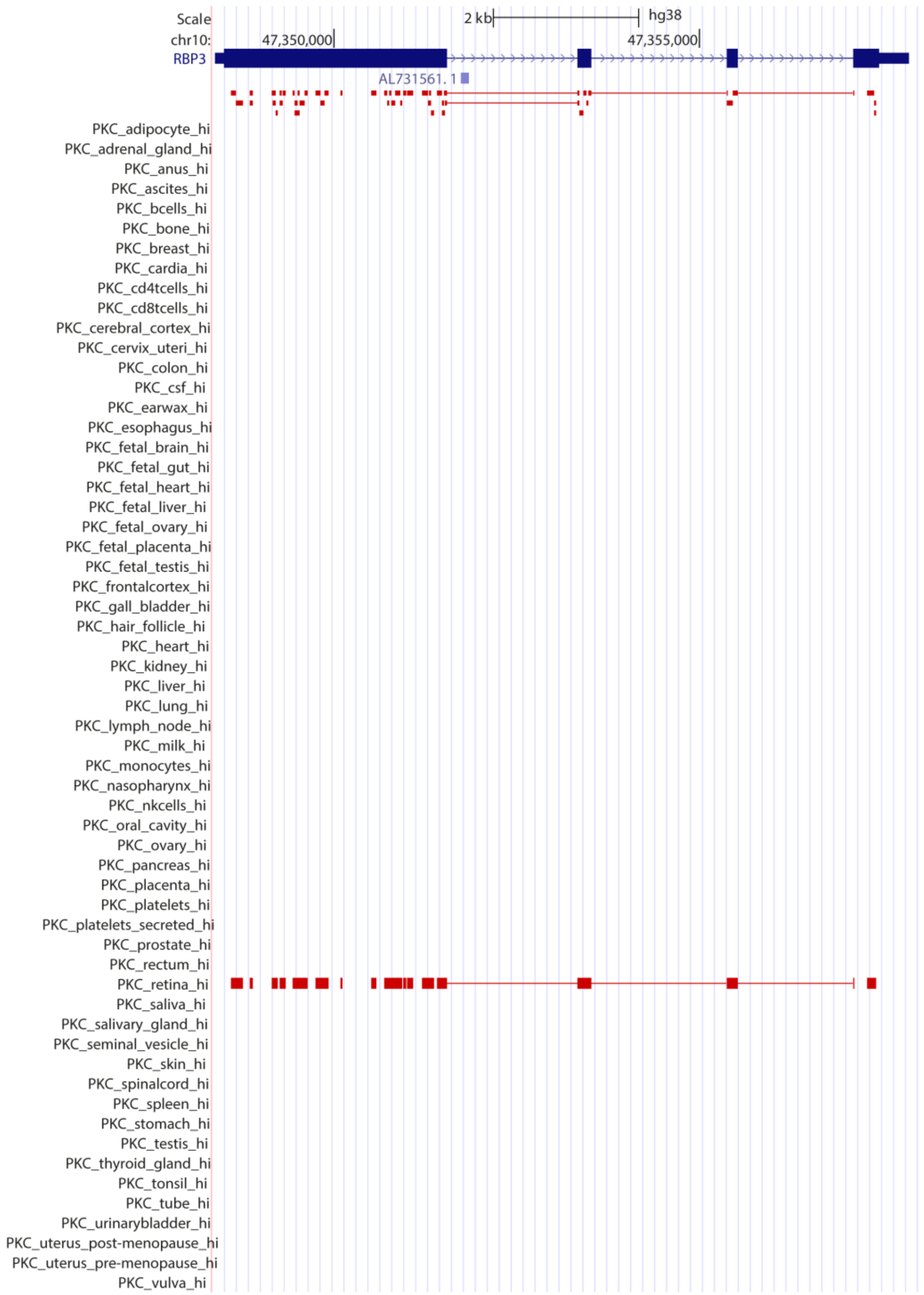

Figure S3. Track-hub visualization for peptides of RBP3, Related to STAR Methods. Visualization of track-hub generator output for the reanalyzed draft human proteome maps in the UCSC genome browser for the genomic region of *RBP3*. Genomic coordinates are shown as x-axis while tissues within the dataset represent the y-axis. GENCODE (v20) annotation of two transcripts is shown in black at the top. Peptides identified in the whole dataset are shown underneath in red, representing unique mapping to a single transcript, and black, indicating unique mapping to the gene. Peptides identified within single tissues are shown below. All peptides identified in the region were only found in retina (red and black bars in the lower third of screenshot) spanning all three splice junctions showing proteomic support for the annotated gene structure.

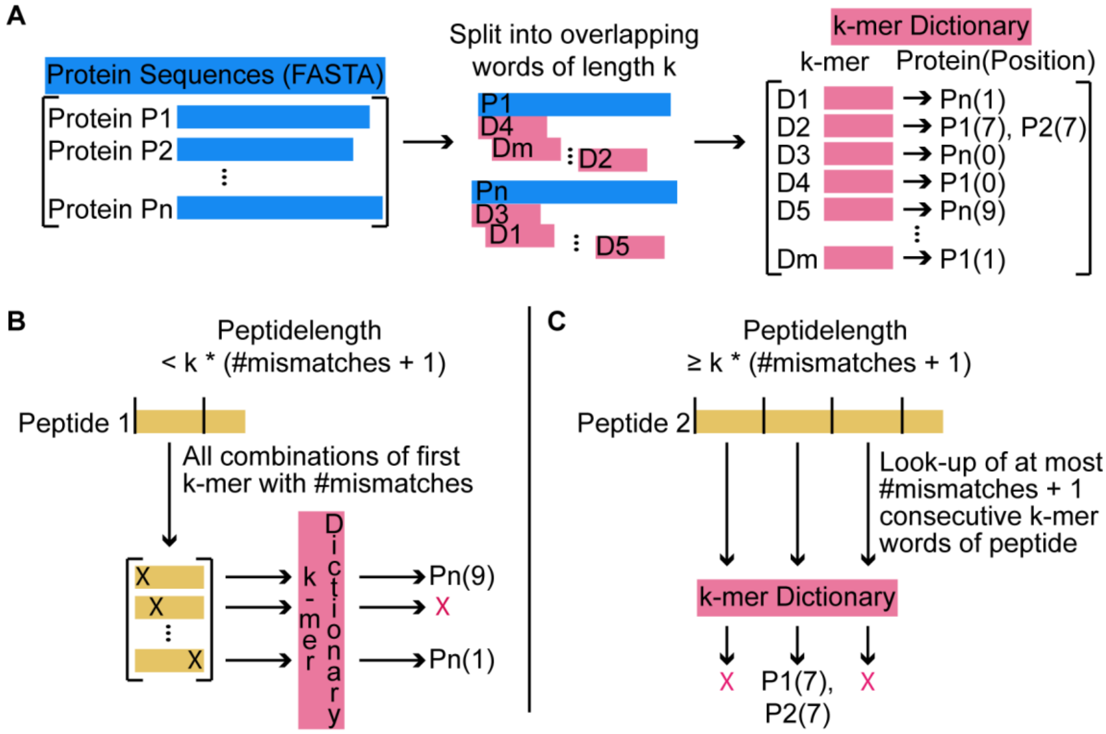

Figure S4. Schematic of algorithm enabling variant mapping, Related to Figure 1. Graphical representation of the initial step in PoGo algorithm that generates and uses an indexed dictionary lookup supporting amino acid variants to identify annotated proteins for a given input peptide sequence and enable genomic mapping. **(A)** The annotated proteins from the FASTA input are indexed through splitting the sequences into words of length k (k-mer) overlapping each other by k-1 amino acids. For each k-mer the originating proteins and the positions of the word within the protein sequence are stored. **(B)** Lookup procedure for input peptides shorter than k times one plus the number of mismatches. The first word of length k from the peptide sequence is used to generate combinations of allowed mismatches within the word. Each new k-mer then is looked up in the dictionary to retrieve associated proteins and start positions. **(C)** For peptides longer than k times one plus the number of mismatches will contain at least one non-overlapping k-mer without a substitution. Therefore the peptide is split into consecutive words of length k. Each word then is used to look up matching proteins in the k-mer dictionary.

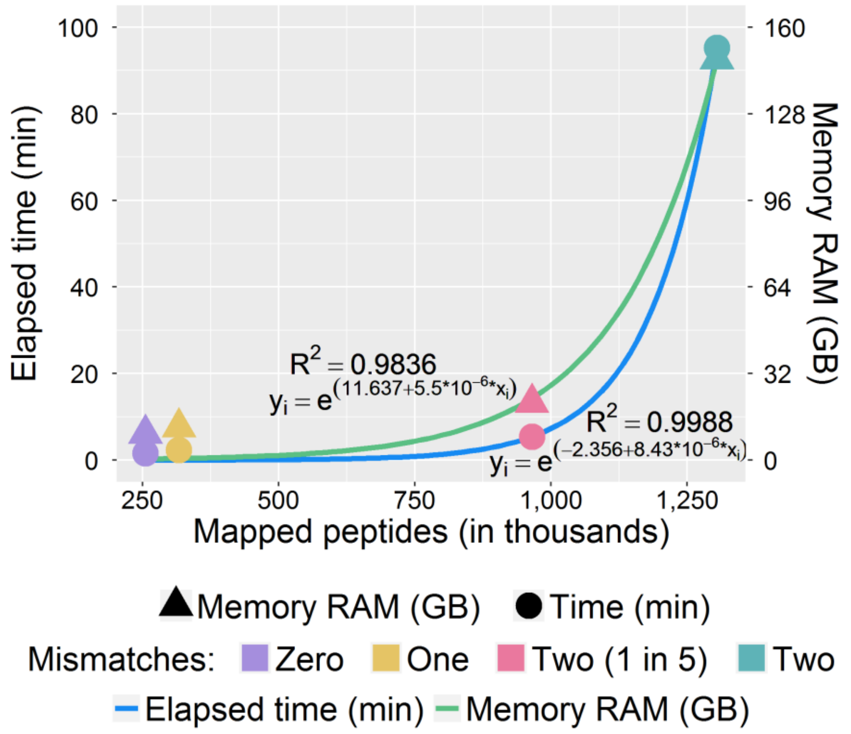

Figure S5. Comparison of runtime, memory and mapped loci for substitution enabled mapping, Related to Figure 1. Comparison of runtime (left side y-axis), number of mapped loci (x-axis), and memory requirements (right side y-axis) across multiple settings for PoGo's unique functionality allowing 0, 1, and 2 mismatches between the reference protein and peptide sequences. The setting allowing 2 mismatches is split into two classes allowing them over the whole peptide length: (i) mismatches have to be at least 5 amino acids apart and (ii) any position within the peptide is allowed to accommodate a mismatch. All three measured variables increase exponentially with the number of mismatches allowed.

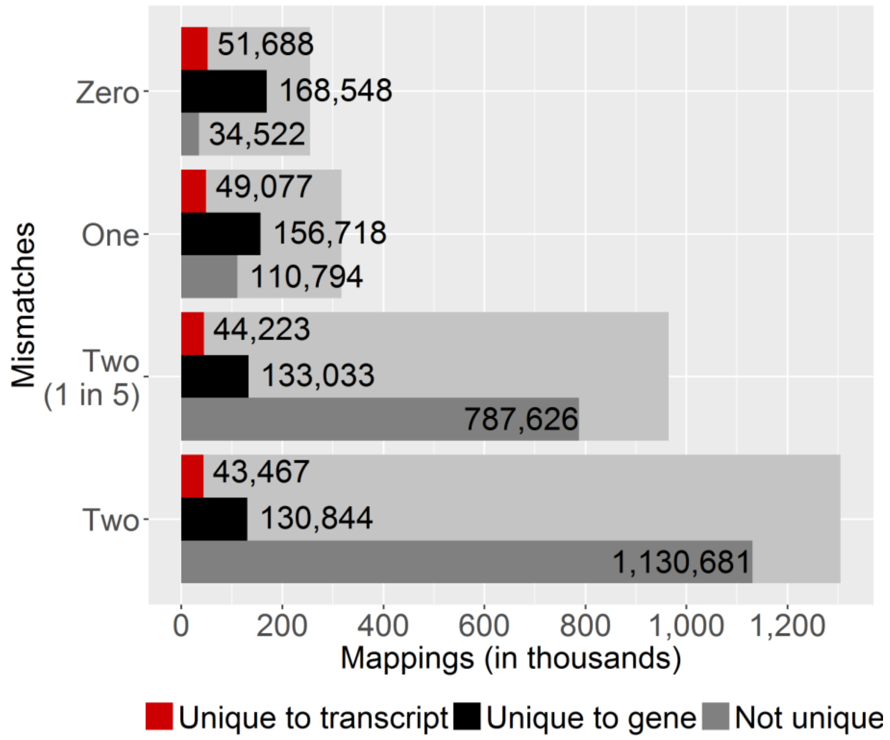

Figure S6. Distribution of uniqueness for substitution enabled mapping, Related to Figure 1. Distribution of mappings between different uniqueness classes over application of PoGo with different settings accounting for 0, 1, and 2 mismatches (y-axis). The setting allowing 2 mismatches is split into two classes allowing them over the whole peptide length: (i) mismatches have to be at least 5 amino acids apart and (ii) any position within the peptide is allowed to accommodate a mismatch. While the overall number of mappings increases exponentially for more allowed mismatches and the number of unique mappings to single transcripts only drops by ~8,000 between 0 and 2 mismatches. The reverse direction for mappings to multiple genes, however, as an exponential function, indicates that small numbers of amino acid substitutions reduce the number of reliable mapping of peptides to unique proteins in a reference database significantly.

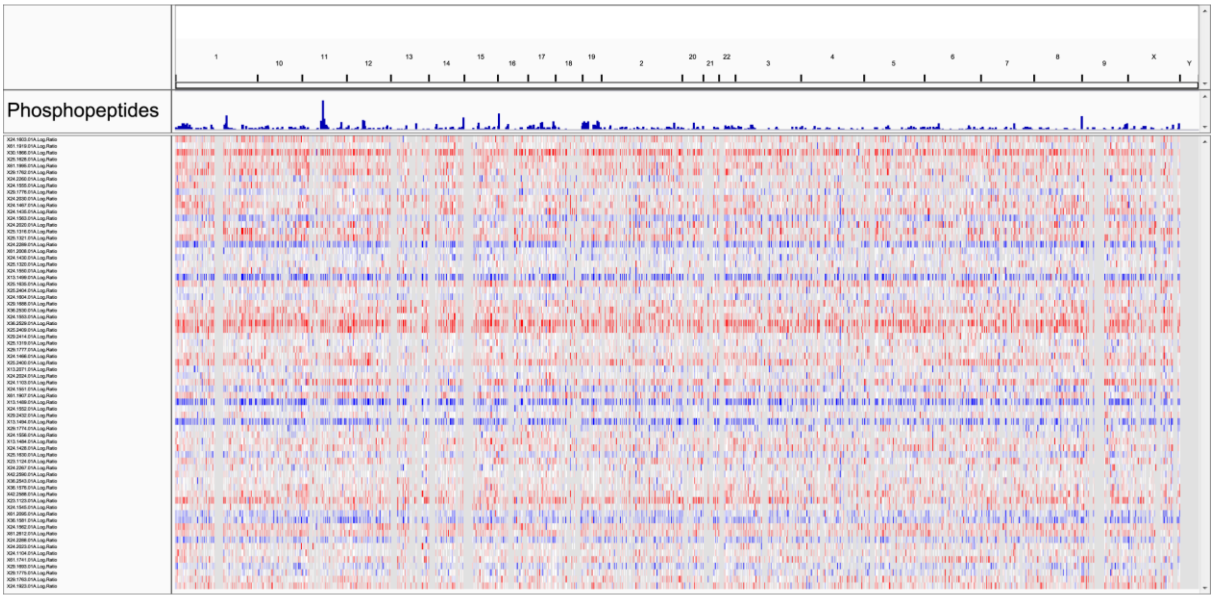

Figure S7. Visualization of phosphoproteome with quantitative features of 69 ovarian cancer samples, Related to Figure 2. The x-axis represents coordinates across the whole human reference genome (GRCh38). The histogram indicates the number of mappings for phosphopeptides per genomic locus. The heat map underneath indicates the log2-fold changes of peptide expression over all samples (y-axis) compared to a pooled reference sample. This visualization through quantitative mapping within PoGo enables comparative analysis on a genome wide scale.

Modification	PSI-MS Name	Color	
Phosphorylation	phospho	red	
Acetylation	acetyl	dark orange	
Amidation	amidated	light orange	
Oxidation	oxidation	yellow	
Methylation	methyl	dark green	
Ubiquitinylation	glygly or gg	light green	
Sulfation	sulfo	light turquoise	
Palmitoylation	palmitoyl	dark turquoise	
Formylation	formyl	dark blue	
Deamidation	deamidated	purple	
Any other post-translational modification		pink	

Table S1. Color coding of multiple post-translational modifications in PoGo output, Related to Figure 2 and STAR Methods. PoGo is capable of mapping post-translational modifications to genomic loci and further uses color coding to distinguish between different modification types. The default color code is shown in the table.

Data S1. PoGo test procedures and files, Related to STAR Methods. Small scale test dataset, PoGo binaries and graphical user interface with detailed step by step instructions.
