# Supplementary material for: Fast, Quantitative and Variant Enabled Mapping of Peptides to Genomes
Source: Cell Syst. 2017 Aug 23;5(2):152–156.e4. doi: 10.1016/j.cels.2017.07.007 (PMC5571441; doi:10.1016/j.cels.2017.07.007)
Supplement: Data S1. PoGo Test Procedures and Files, Related to STAR Methods [file mmc2.zip › Data S1/PoGo_Testprocedures.pdf]

PoGo

Datasets for PoGo tests

For testing we provide a collection of 17 unique peptides covering all mapping capabilities of PoGo including multiple mappings, post-translational modifications, high complexity regions and multiple splicing events.

Procedures for testing PoGo

Executables of PoGo are included in this download in the folder 'Executables' for linux, Windows and Mac. The C++ source code is made available through https://github.com/cschlaffner/PoGo.git. To test the software on your system please follow the instructions below. For a full download with all required files and an additional larger test dataset please go to www.sanger.ac.ku/science/tools/pogo. The download link is located at the bottom of the section 'Learn' and skip step 1.

1. Download annotation and translated sequences for human from GENCODE version 25. Go to www.gencodegenes.org/releases/25.html and download the GTF file containing 'Comprehensive gene annotation' and the 'Protein-coding transcript translation sequences' as Fasta file. Store and unzip both files into the folder ${POGO_DIR}/Testfiles/input/

2. Navigate to the unzipped PoGo directory and into the folder matching your operating system.

   cd ${POGO_DIR}/Executables/${OS_DIR}

3. Execute the following command to generate gtf, gct, bed and ptmbed output for either of the test files Testpeptides_small.txt and Testpeptides_expertimental.txt referred to as ${TestPeptides}

   Linux/Unix
   ./PoGo –fasta ../../Testfiles/input/gencode.v25.pc_translations.fa –gtf ../../Testfiles/input/gencode.v25.annotation.gtf –in ../../Testfiles/input/${TestPeptides}

   Windows
   .\PoGo.exe –fasta ..\..\Testfiles\input\gencode.v25.pc_translations.fa –gtf ..\..\Testfiles\input\gencode.v25.annotation.gtf –in ..\..\Testfiles\input\${TestPeptides}

4. You can compare the generated output with the true mappings provided in the directory ${POGO_DIR}/Testfiles/correct_output/ either through direct comparison of files or via loading the generated BED and/or GTF files with the corresponding correct outputs into a genome browser. Here the example is shown for visualisation in the UCSC genome browser.

   To load the data into the browser please follow these steps:
   a. Go to https://genome.ucsc.edu and navigate to 'My Data' -> 'Custom Tracks'.
   b. After clicking 'Choose File' select the file you want to upload and submit via the 'Submit' button.
   c. You will be redirected to the 'Manage Custom Tracks' webpage.
   d. Repeat with the corresponding file from the correct_output folder and proceed from the 'Manage Custom Tracks' page by selecting 'Genome Browser' and confirm ('go').
   e. Now you can browse the peptides mapped to their genomic loci on the reference genome.

To test the mapping with allowed amino acid substitutions please follow one of the following commands and load the result files in a genome browser as described in step 4.

./PoGo –fasta ../../Testfiles/input/gencode.v25.pc_translations.fa –gtf ../../Testfiles/input/gencode.v25.annotation.gtf –in ../../Testfiles/input/Testfile_small.txt –mm 1

./PoGo –fasta ../../Testfiles/input/gencode.v25.pc_translations.fa –gtf ../../Testfiles/input/gencode.v25.annotation.gtf –in ../../Testfiles/input/Testfile_small.txt –mm 2 –mmode true

./PoGo –fasta ../../Testfiles/input/gencode.v25.pc_translations.fa –gtf ../../Testfiles/input/gencode.v25.annotation.gtf –in ../../Testfiles/input/Testfile_small.txt –mm 2

Procedures for testing PoGo

A ready to use JAR file of PoGoGUI is included in this download in the folder 'Executables'. The java source code is made available through https://github.com/cschlaffner/PoGoGUI.git. To test the software on your system please follow the instructions below. For a full download with all required files and an additional larger test dataset please go to www.sanger.ac.ku/science/tools/pogo. The download link is located at the bottom of the section 'Learn'. Step 1 can be skipped if you have tested PoGo with the above instructions before.

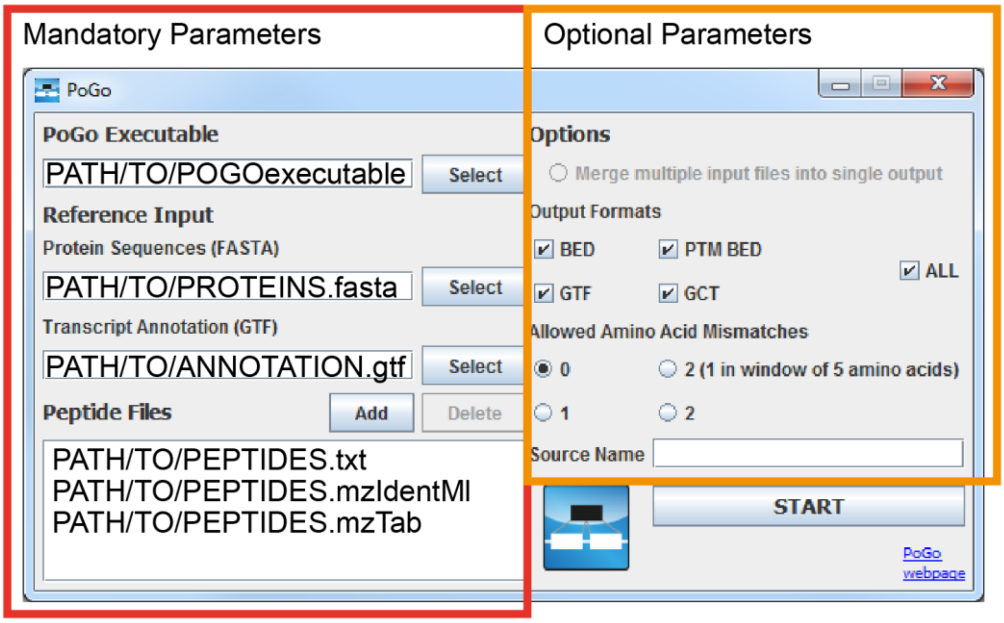

1. Download annotation and translated sequences for human from GENCODE version 25. Go to www.gencodegenes.org/releases/25.html and download the GTF file containing 'Comprehensive gene annotation' and the 'Protein-coding transcript translation sequences' as Fasta file. Store and unzip both files into the folder ${POGO_DIR}/Testfiles/input/

2. Navigate to the unzipped PoGo directory and into the folder containing executables.

   cd ${POGO_DIR}/Executables/

3. Execute the following command to start the GUI

   java –jar ${POGO_DIR}/Executables/PoGoGUI.jar

4. In the user interface select the PoGo executable via the ‘Select’ button.

5. Select the translated protein coding sequence file (FASTA) and the transcript annotation file (GTF) through the respective ‘Select’ buttons.

6. Add the input files in ${POGO_DIR}/Testfiles/ through the ‘Add’ button or through drag and drop into the text field.

7. Through clicking the Start button the PoGo is started

8. After PoGo has finished you can compare the generated output with the true mappings provided in the directory ${POGO_DIR}/Testfiles/correct_output/ either through direct comparison of files or via loading the generated BED and/or GTF files with the corresponding correct outputs into a genome browser. Here the example is shown for visualisation in the UCSC genome browser.

   To load the data into the browser please follow these steps:

   a. Go to https://genome.ucsc.edu and navigate to ‘My Data’ -> ‘Custom Tracks’.
   b. After clicking ‘Choose File’ select the file you want to upload and submit via the ‘Submit’ button.
   c. You will be redirected to the ‘Manage Custom Tracks’ webpage.
   d. Repeat with the corresponding file from the correct_output folder and proceed from the ‘Manage Custom Tracks’ page by selecting ‘Genome Browser’ and confirm (‘go’).
   e. Now you can browse the peptides mapped to their genomic loci on the reference genome.

To test the mapping with allowed amino acid substitutions please follow the above commands and select the number of mismatches through clicking the associated ratio button. The result files can be loaded in a genome browser as described in step 4.

Hardware and Software requirements

<u>For test dataset</u>

Hardware: min 2GB RAM, min 2GB free memory

Software:

- any of these operating systems Windows Vista/7/8/8.1/10, Linux or Mac OS
- a text editor
- internet browser
- java 7 or higher

<u>For experimental datasets</u>

Hardware: min 4GB RAM with respect to the number of peptides in the input file (see Table 1), and min 4GB free memory

Software:

- any of these operating systems Windows Vista/7/8/8.1/10, Linux or Mac OS
- a text editor

- internet browser
- -java 7 or higher

Estimates of time required for testing

For test datasets

For the Testfile_small.txt running PoGo and loading the resulting output in a genome browser will take no more than 10 minutes.

For the Testfile_experimental.txt running PoGo will take no more than 15 minutes.

For experimental datasets

For experimental datasets the runtime of PoGo is dependent of the selected setting and the number of peptides in the input file. Please refer to Table 1 for estimates of runtime and required memory. Larger numbers of peptides will result in output files larger than 20MB. In these cases the output BED and GTF files are too big to be loaded directly into an online genome browser please follow the instructions for generating track hubs with 'TrackHubGenerator' (http://sanger.ac.uk/science/tools/trackhub-generator).

Table 1 Runtime and required memory (RAM) for PoGo execution across different settings for inclusion of mismatches and depending on number of peptides in the input file.

Mismatch parameter settings	~1,500 peptides	~250,000 peptides
-mm 0	< 5 min / < 4GB	< 5 min / min 10 GB
-mm 1	~ 5 min / < 4 GB	~ 5 min / min 16 GB
-mm 2 –mmode true	< 15 min / min 6 GB	< 20 min / min 32 GB
-mm 2 –mmode false	~ 1.5 h / min 64 GB	~ 2 h / min 160 GB
